# Sugar-Sweeten Beverage Consumption Is Associated With More Obesity and Higher Serum Uric Acid in Chinese Male Gout Patients With Early Onset

**DOI:** 10.3389/fnut.2022.916811

**Published:** 2022-07-12

**Authors:** Qian-Hua Li, Yao-Wei Zou, Shu-Yan Lian, Jin-Jian Liang, Yu-Fei Bi, Chao Deng, Ying-Qian Mo, Kui-Min Yang, Lie Dai

**Affiliations:** ^1^Department of Rheumatology, Sun Yat-sen Memorial Hospital, Sun Yat-sen University, Guangzhou, China; ^2^Department of Rheumatology, Shenshan Medical Center, Memorial Hospital of Sun Yat-sen University, Shanwei, China; ^3^Department of Rheumatology, Qilu Hospital, Shandong University, Qingdao, China; ^4^Department of Rheumatology, Shenzhen Third People’s Hospital, The Second Affiliated Hospital, Southern University of Science and Technology, Shenzhen, China

**Keywords:** early onset gout, food frequency questionnaire, sugar-sweeten beverages, obesity, serum urate

## Abstract

**Background:**

Early onset gout has received increasing interest from researchers. Previous studies have reported that serum urate (sUA) levels and prevalence of obesity are higher in early onset gout patients than in later-onset gout patients. We explored the dietary habits of early onset and later-onset gout patients and their association with clinical features.

**Materials and Methods:**

Gout patients completed a 10-item food frequency questionnaire. Early onset gout patients were defined as gout onset before the age of 40, and onset after age 40 was classified as later-onset. Associations between dietary factors, obesity, and sUA level of ≥600 μmol/L were assessed using logistic regression.

**Results:**

Among the 655 gout patients, 94.6% were males, and 59.1% presented with early onset gout. All early onset patients were males. sUA level was significantly higher in the early onset group than in the later-onset group (550.7 vs. 513.4 μmol/L). The proportion of patients with a sUA level of ≥ 600 μmol/L (40.3 vs. 26.2%) and obesity (27.6 vs. 10.7%) was higher in the early onset group than in the later-onset group (all *p* < 0.05). The early onset group consumed more red meat (101–200 g/day: 43.6 vs. 26.0%), sugar-sweetened beverages (>4 times/week: 27.9 vs. 7.7%), and milk and milk products (1–2 times/week: 28.5 vs. 16.6%), but less alcohol (>84 g/day: 8.5 vs. 21.5%) and tea (>4 times/week: 35.7 vs. 52.4%; all *p* < 0.05). Sugar-sweetened beverage intake was positively correlated with sUA level of ≥600 μmol/L (compared with <once/week [reference], >4 times/week: adjusted odds ratio = 2.2, 95% confidence interval: 1.4, 3.7) and obesity (compared with <once/week [reference], >4 times/week: adjusted odds ratio = 2.2, 95% confidence interval: 1.2, 3.7). These correlations remained significant for early onset gout patients.

**Conclusion:**

Sugar-sweetened beverage intake replaced alcohol as the main dietary risk factor for gout in early onset patients, and this change was associated with a greater prevalence of obesity and higher sUA level. Clinicians should provide specific dietary education for different generations of gout patients. The epidemic of sugar-sweetened beverage consumption should be considered for the development of public health policies for the prevention of gout.

## Introduction

Gout is a chronic disease caused by long-term hyperuricemia, which leads to the deposition of monosodium urate crystals in the joints, adjacent structures, and other tissues. Genetic and environmental factors are involved in the development of hyperuricemia. Genetic analysis has identified approximately 30 loci associated with hyperuricemia (1). A recent meta-analysis found that single nucleotide polymorphisms contribute to nearly one-quarter of the variance of serum urate (sUA) level (2). Dietary factors, such as alcohol, red meat, and seafood consumption, are well-established risk factors of hyperuricemia and gout flares (3). Moreover, fructose intake is strongly positively correlated with sUA level (4) and gout risk (5, 6). Fructose is also an important risk factor for obesity and type 2 diabetes mellitus (T2DM), which are common comorbidities in gout patients (7).

The prevalence of gout has continued to rise globally (8). Approximately 40 years ago, gout was considered a rare disease in China; no gout was reported in a survey on health screening across four large cities in 1980 (9). However, a recent meta-analysis has shown that the prevalence of gout has increased to 1.4% between 2011 and 2014 in China (10), and a recent cross-sectional study found that nearly one-third of all college students are diagnosed with hyperuricemia in China (11). Several studies to date have focused on early onset gout (11–15). Nearly 60% of Chinese gout patients at a tertiary hospital in Beijing experienced an initial gout flare before the age of 40 between 2008 and 2014 (13). Younger gout patients are characterized by a higher sUA level and body mass index (BMI) than those of older patients (13, 15).

Genetic variants contribute to higher sUA levels in early onset gout patients. Among the genetic variants that are related to heightened sUA levels, the ABCG2 rs2231142 T-allele is associated with gout onset before the age of 40 (16). Although dietary factors are well-established risk factors for gout, differences in dietary habits between early onset and later-onset gout patients have not been reported. Furthermore, the influence of dietary factors on the clinical features of early onset gout patients has also yet to be ascertained. Therefore, we conducted a questionnaire survey on food intake frequency in Chinese gout patients to evaluate dietary habits and clinical features in gout patients.

## Materials and Methods

### Patients and Grouping

Consecutive patients who met the 2015 gout classification criteria (17) were recruited from August 2017 to July 2021 from the Department of Rheumatology, Sun Yat-sen Memorial Hospital. Exclusion criteria were as follows: patients had already received urate-lowering therapy, pyrazinamide, azathioprine, or cyclosporine within the 1 month before study enrolment; gout onset before the age of 16, and gout secondary to single gene disorders, malignancy, and hematological proliferative diseases. Patients who experienced an initial gout attack before the age of 40 were classified as early onset patients (16), and all other patients were classified as later-onset. This study was conducted in accordance with the Declaration of Helsinki and was approved by the Ethics Committee of Sun Yat-sen Memorial Hospital (SYSEC-KY-KS-022). All patients provided written informed consent before participating in the study.

### Clinical and Laboratory Assessments

Demographic and disease assessment data were collected from all patients. Gout assessment included age of onset, disease duration, counts of ever-involved joints, tophi, and nephrolithiasis, gout attack times during the preceding year, and family history of gout. Subjects were categorized by BMI as obese (BMI ≥ 28 kg/m^2^), overweight (24 kg/m^2^ ≤ BMI < 28 kg/m^2^), normal weight (18.5 kg/m^2^ ≤ BMI < 24 kg/m^2^), and underweight (BMI < 18.5 kg/m^2^) (18). Central obesity was defined as a waist circumstance of ≥90 cm for men and ≥85 cm for women (18).

Overnight fasting blood samples were collected from basilic vein to measure sUA level, serum creatinine (SCr), plasma glucose (FPG), and lipid profiles using the Hitachi 7600-110 Chemistry Autoanalyzer. Ultrasonography was used to evaluate fatty liver disease and nephrolithiasis. Comorbidities of the gout patients were recorded, which included obesity, hypertension, T2DM, dyslipidemia, metabolic syndrome, coronary heart disease, chronic kidney disease (CKD), urolithiasis, and fatty liver disease. Diagnoses of hypertension, T2DM, dyslipidemia, and metabolic syndrome were made according to the latest local guidelines (19–21). Estimated glomerular filtration rate (eGFR) was calculated using the following equation: eGFR (ml/min/1.73 m^2^) = 175×SCr (mg/dl)^−1.234^×age^−0.179^×0.79 (if female) (22). CKD was defined as an eGFR of <60 ml/min/1.73 m^2^.

### Food Frequency Questionnaire and Judgment Criteria

A simplified food frequency questionnaire was adapted from the food frequency questionnaire of the China National Nutrition and Health Survey (23). Foods and food groups were selected according to the 2012 American College of Rheumatology guidelines for the management of gout (24) and included animal offal, red meat, seafood, alcohol, sugar-sweetened beverages (SSB), coffee, and milk and milk products. SSB included soda, fruit juice, fruit-flavored drink, cordials, sport drinks and herbal tea which were sweetened by high fructose corn syrup or sucrose. Hotpot, slow-cooking soup, and tea were also included because these popular Chinese foods may influence sUA level. The final questionnaire comprised 10 items. All patients completed the questionnaire in approximately 10–15 min.

Trained investigators administered the questionnaire to outpatients in the clinical room and inpatients at their bedside. Each item was explained to the patients. Patients completed the questionnaire on their own. After the patient completed the questionnaire, the investigator reviewed the questionnaire and confirmed the item’s answer with the patient. Because patients are prone to change their dietary habits after being diagnosed with gout, participants were informed to report their average intake frequency during the 1 year before the first gout flare. Questionnaires with a minimum of eight answered items were regarded as valid for statistical analysis.

For alcohol intake, patients were asked about the frequency, categories (beer, wine, or spirits), ethanol concentration, and volume consumed. A standard drink was used to evaluate alcohol intake. A standard drink contains approximately 14 g of alcohol, which is equivalent to 497 ml of 3.5% beer, 145 ml of 12% wine, or 44 ml of 40% spirits (25). Alcohol intake was calculated by multiplying the consumption of alcoholic drinks by the corresponding ethanol content. Alcohol intake in this study was divided into three categories: 0–28 g/day, 29–84 g/day, and >84 g/day. Daily consumption of red meat was categorized as less than 100 g/day, 101–200 g/day, and >200 g/day. Consumption of animal offal, seafood, hotpot, slow-cooking soup, SSB, tea, coffee, and milk and milk products was assessed by weekly frequency without portion sizes. Intake of these eight items was categorized as <once/week), 1–2 times/week, 3–4 times/week, and >4 times/week.

### Statistical Analysis

Data were analyzed using SPSS Statistics for Windows 20.0 (IBM, Armonk, NY, United States). Categorical variables are presented as frequencies and percentages. Continuous variables are presented as means and standard deviations (SD) for normal distributed data or medians and interquartile ranges (IQRs) for data with a skewed distribution. The normal distribution of continuous variables was evaluated by Kolmogorov–Smirnov test. Differences in continuous variables between the two groups were tested using two-samples *t*-tests for the normally distributed variables and the Mann–Whitney test for non-normally distributed variables. A chi-square test was used for categorical variables. Missing data were addressed using pairwise deletion.

Correlations between dietary factors and obesity and sUA level of ≥600 μmol/L were evaluated using univariate logistic regression analyses. Further multivariate logistic regression analyses were applied to confirm the correlation between dietary factors and obesity after adjusting for potential confounding factors, which included duration of gout, family history, eGFR, comorbidities (i.e., hypertension, T2DM, dyslipidemia, metabolic syndrome, fatty liver disease, and coronary heart disease, and other dietary factors that were significant at *p* < 0.05 in the univariate logistic regression analysis. The correlation between dietary factors and sUA level ≥600 μmol/L was adjusted by the above confounding factors, obesity, and current medications (i.e., aspirin, diuretics, and lipid-lowering medication including atorvastatin and fenofibrate). The above correlations were performed both in male gout patients and early onset gout patients. All tests were conducted using a two-tailed 5% significance level.

## Results

### Demographic Characteristics of Gout Patients

**FIGURE 1:**
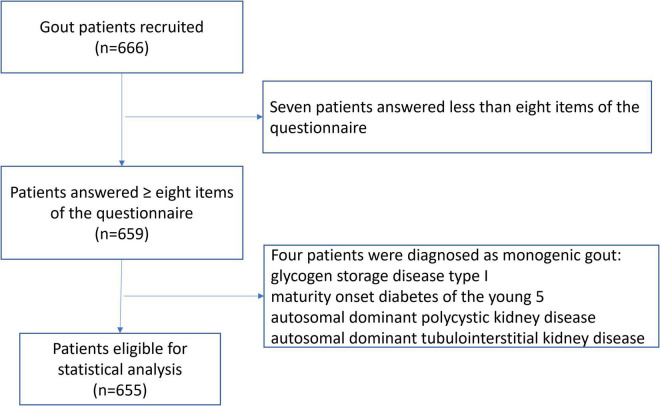
Flow diagram showing the number of included and excluded gout patients.

A total of 666 patients were recruited. Eleven patients were excluded, which included seven patients who answered fewer than eight items of the questionnaire and four patients who were re-diagnosed with monogenic gout (Figure 1). This resulted in 655 patients being valid for statistical analysis. Among these, 620 (94.6%) were males with a mean age of 42.7 ± 14.2 years and a mean age of gout onset of 37.5 ± 13.3 years. There were 35 (5.3%) females with gout, with a mean age of 63.2 ± 12.4 years and a mean age of gout onset of 58.5 ± 12.1 years. There were 161 (24.6%) patients who presented with tophi. The median gout duration (follow-up time) was 4 (2, 7) years. The mean sUA level was 537.1 ± 133.7 μmol/L, and 231 (35.3%) patients had an sUA level of over 600 μmol/L. Detailed demographic characteristics are shown in Table 1.

**TABLE 1:** Demographic and clinical characteristics of 655 gout patients.

Characteristics	All patients (*n* = 655)	Male patients (*n* = 620)	Female patients (*n* = 35)	*P*
Age, years, mean ± SD	43.8 ± 14.8	42.7 ± 14.2	63.2 ± 12.4	**<0.001**
Onset age, years, mean ± SD	38.6 ± 14.0	37.5 ± 13.3	58.5 ± 12.1	**<0.001**
Gout duration, years, median (IQR)	4 (2, 7)	4 (2, 7)	3 (1, 7)	0.516
Count of ever involved joints, median (IQR)	4 (2, 7)	4 (2, 6)	4 (2, 8)	0.413
Flare times in the last year, median (IQR)	4 (2, 10)	4 (2, 10)	6 (2, 24)	0.212
Family history of gout, *n* (%)	235 (35.9)	219 (35.3)	16 (45.7)	0.277
Tophi, *n* (%)	161 (24.6)	151 (24.4)	10 (28.6)	0.687
sUA, μmol/L, mean ± SD	537.1 ± 133.7	536.7 ± 134.0	544.3 ± 129.9	0.745
sUA ≥ 600 μmol/L	231 (35.3)	214 (35.0)	14 (40.0)	0.587
SCr, μmol/L, mean ± SD	99.1 ± 23.9	99.3 ± 23.7	94.7 ± 27.9	0.341
eGFR, ml⋅min^–1^⋅1.73 m^–2^, mean ± SD	82.6 ± 24.4	83.4 ± 24.1	68.2 ± 25.0	**<0.001**
CKD, *n* (%)	65 (9.9)	50 (8.1)	15 (42.9)	**<0.001**
Urolithiasis, *n* (%)	167 (25.5)	160 (25.8)	7 (20.0)	0.552
BMI, kg/m^2^, mean ± SD	25.4 ± 3.6	25.5 ± 3.6	25.3 ± 4.0	0.782
Obese, *n* (%)	141 (21.5)	132 (21.3)	9 (25.7)	0.171
Overweight, n (%)	283 (43.2)	269 (43.4)	14 (40.0)	
Normal BMI, *n* (%)	221 (33.7)	211 (34.0)	10 (28.6)	
Underweight, *n* (%)	10 (1.5)	8 (1.3)	2 (5.7)	
Waist circumstance, cm, mean ± SD	90.9 ± 9.7	91.0 ± 9.6	89.0 ± 10.2	0.241
Central obesity, *n* (%)	370 (56.5)	347 (56.0)	23 (65.7)	0.296
Dyslipidemia, *n* (%)	413 (63.12)	390 (62.9)	23 (65.7)	0.858
Hypercholesterolemia, *n* (%)	143 (21.8)	129 (20.8)	14 (40.0)	**0.011**
Hypertriglyceridemia, *n* (%)	197 (30.1)	185 (29.8)	12 (34.3)	0.705
LDL-C hyperlipidemia, *n* (%)	148 (22.6)	135 (21.8)	13 (37.1)	**0.040**
HDL-C hypolipidemia, *n* (%)	179 (27.3)	170 (27.4)	9 (25.7)	0.850
Metabolic syndrome, *n* (%)	302 (46.1)	282 (45.5)	20 (57.1)	0.220
Fatty liver disease, *n* (%)	316 (48.2)	301 (48.5)	15 (42.9)	0.603
T2DM, *n* (%)	80 (12.2)	66 (10.6)	14 (40.0)	**<0.001**
Hypertension, *n* (%)	278 (42.4)	256 (41.3)	22 (62.9)	**0.012**
Coronary heart disease, *n* (%)	19 (2.9)	14 (2.32)	5 (14.3)	**<0.001**

*Bold values mean the difference is statistically significant.*

**FIGURE 2:**
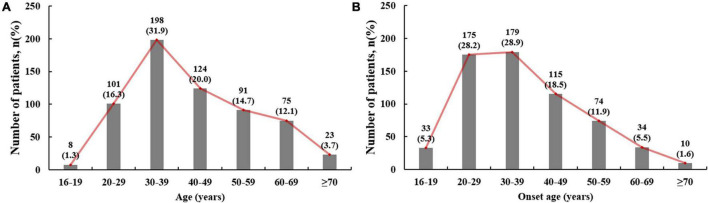
Distribution of age **(A)** and onset age **(B)** in male gout patients.

The highest proportion of patients had an onset age of 30–39 years (28.9%), followed by 20–29 years (28.1%) and 40–49 years (18.8%; Figure 2). There were 387 (59.1%) patients in the early onset group, with a mean onset age of 28.8 ± 6.0 years. Because all patients with early onset gout were males, subsequent statistical analyses included only the male patients.

### Clinical Characteristics of Early Onset Gout Patients

Compared with later-onset male patients, early onset male gout patients presented with significantly higher sUA level (550.7 ± 135.2 μmol/L vs. 513.4 ± 129.0 μmol/L, *p* < 0.05), and had a higher proportion of patients who had an sUA level of ≥600 μmol/L (40.3 vs. 26.2%, *p* < 0.05). However, eGFR was significantly higher in the early onset group than that in the later-onset group (86.9 ± 16.6 ml/min/1.73 m^2^ vs. 77.7 ± 32.3 ml/min/1.73 m^2^, *p* < 0.05; Table 2). Compared with later-onset male patients, early onset male gout patients presented with a higher BMI (26.0 ± 3.9 kg/m^2^ vs. 24.6 ± 2.8 kg/m^2^; *p* < 0.001) and a higher prevalence of obesity (27.6 vs. 10.7%, *p* < 0.001; Table 2). Male gout patients with a later onset showed more hypertension (57.1 vs. 31.8%, *p* < 0.05), had higher rates of metabolic syndrome (51.5 vs. 41.9%, *p* < 0.05), T2DM (17.2 vs. 6.7%, *p* < 0.05), and coronary heart disease (5.6 vs. 0.3%, *p* < 0.05), and a higher count of ever-involved joints (4 vs. 3, *p* < 0.05) than those of early onset patients.

**TABLE 2:** Comparison of demographic and clinical characteristics between male gout patients with early onset and later-onset.

Characteristics	Early onset group (*n* = 387)	Later-onset group (*n* = 233)	*P*
Age, years, mean ± SD	33.9 ± 7.8	57.3 ± 9.5	**<0.001**
Onset age, years, mean ± SD	28.8 ± 6.0	51.8 ± 9.0	**<0.001**
Gout duration, years, median (IQR)	3 (2, 7)	4 (2, 8)	0.070
Count of ever involved joints, median (IQR)	3 (2, 6)	4 (2, 8)	**0.002**
Flare times in the last year, median (IQR)	4 (2, 10)	4 (2, 12)	0.480
Family history of gout, *n* (%)	143 (37.0)	76 (32.6)	0.298
Tophi, *n* (%)	81 (20.9)	70 (30.0)	**0.012**
sUA, μmol/L, mean ± SD	550.7 ± 135.2	513.4 ± 129.0	**0.001**
sUA ≥ 600 μmol/L, *n* (%)	156 (40.3)	61 (26.2%)	**<0.001**
SCr, μmol/L, mean ± SD	97.6 ± 20.7	102.3 ± 27.8	**0.026**
eGFR, ml⋅min^–1^⋅1.73 m^–2^, mean ± SD	86.9 ± 16.6	77.7 ± 32.3	**<0.001**
CKD, *n* (%)	18 (4.7)	32 (13.7)	**<0.001**
Urolithiasis, *n* (%)	80 (20.7)	80 (34.3)	**<0.001**
BMI, kg/m^2^, mean ± SD	26.0 ± 3.9	24.6 ± 2.8	**<0.001**
Obese, *n* (%)	107 (27.6)	25 (10.7)	**<0.001**
Overweight, *n* (%)	161 (41.6)	108 (46.4)	
Normal BMI, *n* (%)	113 (29.2)	98 (42.1)	
Underweight, *n* (%)	6 (1.5)	2 (0.9)	
Waist circumstance, cm, mean ± SD	91.2 ± 10.5	90.7 ± 8.1	0.450
Central obesity, *n* (%)	222 (57.4)	125 (53.6)	0.404
Dyslipidemia, *n* (%)	238 (61.5)	152 (65.2)	0.391
Hypercholesterolemia, *n* (%)	83 (21.4)	46 (19.7)	0.683
Hypertriglyceridemia, *n* (%)	123 (31.8)	62 (26.6)	0.176
LDL-C hyperlipidemia, *n* (%)	82 (21.2)	53 (22.7)	0.688
HDL-C hypolipidemia, *n* (%)	97 (25.1)	73 (31.3)	0.095
Metabolic syndrome, *n* (%)	162 (41.9)	120 (51.5)	**0.020**
Fatty liver disease, *n* (%)	198 (51.2)	103 (44.2)	0.098
T2DM, *n* (%)	26 (6.7)	40 (17.2)	**<0.001**
Hypertension, *n* (%)	123 (31.8)	133 (57.1)	**<0.001**
Coronary heart disease, *n* (%)	1 (0.3)	13 (5.6)	**<0.001**

*sUA, serum uric acid; SCr, serum creatinine; eGFR, estimated glomerular filtration rate; CKD, chronic kidney disease; BMI, body mass index; LDL-C, low-density lipoprotein cholesterol; HDL-C, high-density lipoprotein cholesterol; T2DM, type 2 diabetes mellitus. Bold values mean the difference is statistically significant.*

### Food Intake Frequency in Early Onset Male Gout Patients

The frequency of consumption of each food is shown in Table 3. Among the male gout patients, 13.4% consumed >84 g/day of alcohol, 23.4% ate >200 g/day of red meat, 41.9% drank tea, 20.3% consumed SSB, and 10.2% consumed slow-cooking soup >4 times/week. Less than 10% of patients consumed animal offal, seafood, hotpot, coffee, or milk and milk products >4 times/week.

**TABLE 3:** Comparison of food intake frequency in male gout patients with early onset and later-onset.

Food intake frequency	All male gout patients (*n* = 620)	Early onset group (*n* = 387)	Later-onset group (*n* = 233)	*P*
**Alcohol**				
0–28 g/day, *n* (%)	466 (75.2)	312 (80.6)	154 (66.1)	**<0.001**
29–84 g/day, *n* (%)	71 (11.5)	42 (10.9)	29 (12.4)	
≥84 g/day, *n* (%)	83 (13.4)	33 (8.5)	50 (21.5)	
**Red meat (*n* = 592)**				
Less than 100 g/day, *n* (%)	232 (39.4)	130 (34.8)	102 (47.4)	**<0.001**
101–200 g/day, *n* (%)	219 (37.2)	163 (43.6)	56 (26.0)	
≥200 g/day, *n* (%)	138 (23.4)	81 (21.6)	57 (26.6)	
**Animal offal**				
Less than 1 time/week, *n* (%)	424 (68.4)	255 (65.9)	169 (72.5)	0.190
1–2 times/week, *n* (%)	128 (20.6)	83 (21.4)	45 (19.3)	
3–4 times/week, *n* (%)	42 (6.8)	32 (8.3)	10 (4.3)	
>4 times/week, *n* (%)	26 (4.2)	17 (4.4)	9 (3.9)	
**Seafood**				
Less than 1 time/week, *n* (%)	429 (69.2)	262 (67.7)	167 (71.7)	0.218
1–2 times/week, *n* (%)	108 (17.4)	76 (19.6)	32 (13.7)	
3–4 times/week, *n* (%)	44 (7.1)	24 (6.2)	20 (8.6)	
>4 times/week, *n* (%)	39 (6.3)	25 (6.5)	14 (6.0)	
**Hotpot**				
Less than 1 time/week, *n* (%)	546 (88.1)	341 (88.1)	205 (88.0)	0.993
1–2 times/week, *n* (%)	56 (9.0)	35 (9.0)	21 (9.0)	
3–4 times/week, *n* (%)	11 (1.8)	7 (1.8)	4 (1.7)	
>4 times/week, *n* (%)	7 (1.1)	4 (1.0)	3 (1.3)	
**Slow-cooking soup**				
Less than 1 time/week, *n* (%)	268 (43.2)	158 (40.8)	110 (47.2)	0.261
1–2 times/week, *n* (%)	196 (31.6)	126 (32.6)	70 (30.0)	
3–4 times/week, *n* (%)	93 (15.0)	65 (16.8)	28 (12.0)	
>4 times/week, *n* (%)	63 (10.2)	38 (9.8)	25 (10.7)	
**Sugar-sweeten beverages**				
Less than 1 time/week, *n* (%)	339 (54.7)	155 (40.1)	184 (79.0)	<0.001
1–2 times/week, *n* (%)	83 (13.4)	62 (16.0)	21 (9.0)	
3–4 times/week, *n* (%)	72 (11.6)	62 (16.0)	10 (4.3)	
>4 times/week, *n* (%)	126 (20.3)	108 (27.9)	18 (7.7)	
**Tea**				
Less than 1 time/week, *n* (%)	259 (41.8)	183 (47.3)	76 (32.6)	<0.001
1–2 times/week, *n* (%)	64 (10.3)	42 (10.9)	22 (9.4)	
3–4 times/week, *n* (%)	37 (6.0)	24 (6.2)	13 (5.6)	
>4 times/week, *n* (%)	260 (41.9)	138 (35.7)	122 (52.4)	
**Coffee**				
Less than 1 time/week, *n* (%)	562 (90.6)	346 (89.1)	217 (93.1)	0.346
1–2 times/week, *n* (%)	34 (5.5)	26 (6.7)	8 (3.4)	
3–4 times/week, *n* (%)	7 (1.1)	5 (1.3)	2 (0.9)	
>4 times/week, *n* (%)	17 (2.7)	11 (2.8)	6 (2.6)	
**Milk and milk product (*n* = 595)**				
Less than 1 time/week, *n* (%)	360 (60.8)	217 (57.9)	143 (65.9)	0.004
1–2 times/week, *n* (%)	143 (24.2)	107 (28.5)	36 (16.6)	
3–4 times/week, *n* (%)	40 (6.8)	26 (6.9)	14 (6.5)	
>4 times/week, *n* (%)	49 (8.3)	25 (6.7)	24 (11.1)	

*Time/w: the frequency of dietary intake per week. Bold values mean the difference is statistically significant.*

Compared with the later-onset gout patients, early onset male gout patients consumed more red meat (101–200 g/day: 43.6 vs. 26.0%, *p* < 0.05), SSB (>4 times/week: 27.9 vs. 7.7%, *p* < 0.05), and milk and milk products (1–2 times/week: 28.5 vs. 16.6%, *p* < 0.05), but less alcohol (>84 g/day: 8.5 vs. 21.5%, *p* < 0.05) and tea (>4 times/week: 35.7 vs. 52.4%, *p* < 0.05). There were no significant differences in intake of the other five foods between groups.

### Correlation Between Food Intake Frequency and Serum Urate Level Over 600 μmol/L

For all male gout patients, the risk factors for sUA level over 600 μmol/L included red meat (compared with <100 g/day [reference], >200 g/day: odds ratio [OR] = 2.0, 95% confidence interval [CI]: 1.3, 3.0), animal offal (compared with <once/week [reference], >4 times/week: OR = 3.0, 95% CI: 1.3, 6.8), and SSB consumption (compared with <once/week [reference], >4 times/week: OR = 3.0, 95% CI: 2.0, 4.6; Table 4). For early onset gout patients, the risk factors for sUA level over 600 μmol/L were red meat (compared with <100 g/day [reference], >200 g/day: OR = 1.9, 95% CI: 1.1, 3.4) and SSB consumption (compared with <once/week [reference], >4 times/week: OR = 2.5, 95% CI: 1.5, 4.1). For later-onset gout patients, the correlation between food intake frequency and sUA level over 600 μmol/L weren’t significant (*p* > 0.05). There was a marginal positive correlation between SSB consumption and sUA level over 600 μmol/L (*p* = 0.056).

**TABLE 4:** Univariate correlation between food intake frequency and sUA ≥600 μmol/L in male gout patients.

Food intake frequency	All male gout patients		Early onset gout patients		Later-onset gout patients	
	OR (95% CI)	*P*	OR (95% CI)	P	OR (95% CI)	*P*
**Alcohol**						
0–28 g/day	1	0.974	1	0.320	1	0.270
29–84 g/day	0.9 (0.6, 1.6)		1.4 (0.8, 2.8)		0.4 (0.1, 1.2)	
≥84 g/day	1.0 (0.6, 1.6)		1.5 (0.7, 3.1)		0.9 (0.4, 1.8)	
**Red meat**						
Less than 100 g/day	1	**0.008**	1	**0.043**	1	0.099
101–200 g/day	1.2 (0.8, 1.7)		1.0 (0.6, 1.7)		0.9 (0.4, 2.1)	
≥200 g/day	**2.0 (1.3, 3.0)**		**1.9 (1.1, 3.4)**		2.0 (0.9, 4.1)	
**Animal offal**						
Less than 1 time/week	1	**0.028**	1	0.202	1	0.094
1–2 times/week	0.8 (0.5, 1.2)		0.7 (0.4, 1.2)		1.0 (0.4, 2.1)	
3–4 times/week	1.2 (0.6, 2.2)		1.1 (0.5, 2.3)		0.8 (0.1, 3.7)	
>4 times/week	**3.0 (1.3, 6.8)**		2.0 (0.8, 5.5)		6.0 (1.4, 25.2)	
**Seafood**						
Less than 1 time/week	1	0.851	1	0.843	1	0.501
1–2 times/week	1.1 (0.7, 1.7)		0.8 (0.5, 1.5)		1.6 (0.7, 3.6)	
3–4 times/week	0.9 (0.5, 1.9)		1.2 (0.5, 2.9)		0.8 (0.2, 2.4)	
>4 times/week	1.3 (0.7, 2.6)		1.2 (0.5, 2.6)		1.7 (0.5, 5.4)	
**Hotpot**						
Less than 1 time/week	1	0.276	1	0.393	1	0.362
1–2 times/week	1.1 (0.6, 1.9)		1.1 (0.6, 2.3)		0.9 (0.3, 2.6)	
3–4 times/week	3.4 (1.0, 11.6)		3.8 (0.7, 19.9)		2.9 (0.4, 21.4)	
>4 times/week	1.4 (0.3, 6.5)		0.5 (0.0, 4.9)		5.9 (0.5, 66.2)	
**Slow-cooking soup**						
Less than 1 time/week	1	0.332	1	0.409	1	0.863
1–2 times/week	0.9 (0.6, 1.3)		0.9 (0.5, 1.4)		0.9 (0.4, 1.7)	
3–4 times/week	1.1 (0.7, 1.8)		1.1 (0.6, 2.0)		0.8 (0.3, 2.2)	
>4 times/week	0.6 (0.3, 1.1)		0.5 (0.3, 1.2)		0.6 (0.2, 1.8)	
**Sugar-sweeten beverages**						
Less than 1 time/week	1	**<0.001**	1	**0.002**	1	0.056
1–2 times/week	1.4 (0.8, 2.3)		1.3 (0.7, 2.4)		0.7 (0.2, 2.4)	
3–4 times/week	1.4 (0.8, 2.3)		0.9 (0.4, 1.6)		5.0 (1.0, 18.2)	
>4 times/week	**3.0 (2.0, 4.6)**		**2.5 (1.5, 4.1)**		2.6 (0.9, 7.1)	
**Tea**						
Less than 1 time/week	1	0.649	1	0.779	1	0.825
1–2 times/week	0.7 (0.4, 1.3)		0.8 (0.4, 1.5)		0.6 (0.2, 2.1)	
3–4 times/week	0.8 (0.4, 1.7)		0.7 (0.3, 1.7)		1.2 (0.3, 4.5)	
>4 times/week	0.9 (0.6, 1.2)		0.9 (0.6, 1.5)		1.0 (0.5, 2.0)	
**Coffee**						
Less than 1 time/week	1	0.429	1	0.310	1	0.193
1–2 times/week	0.7 (0.3, 1.4)		0.5 (0.2, 1.3)		1.0 (0.2, 5.1)	
3–4 times/week	2.5 (0.5, 11.1)		2.1 (0.4, 12.8)		3.0 (0.2, 49.1)	
>4 times/week	1.3 (0.5, 3.5)		0.5 (0.1, 2.0)		6.0 (1.0, 33.9)	
**Milk and milk product**						
Less than 1 time/week	1	0.895	1	0.812	1	0.918
1–2 times/week	1.1 (0.7, 1.7)		1.1 (0.7, 1.7)		0.9 (0.4, 2.0)	
3–4 times/week	1.2 (0.6, 2.4)		1.3 (0.6, 3.0)		1.1 (0.3, 3.6)	
>4 times/week	1.0 (0.5, 1.9)		1.4 (0.6, 3.2)		0.7 (0.2, 2.0)	

*Bold values mean the difference is statistically significant.*

**FIGURE 3:**
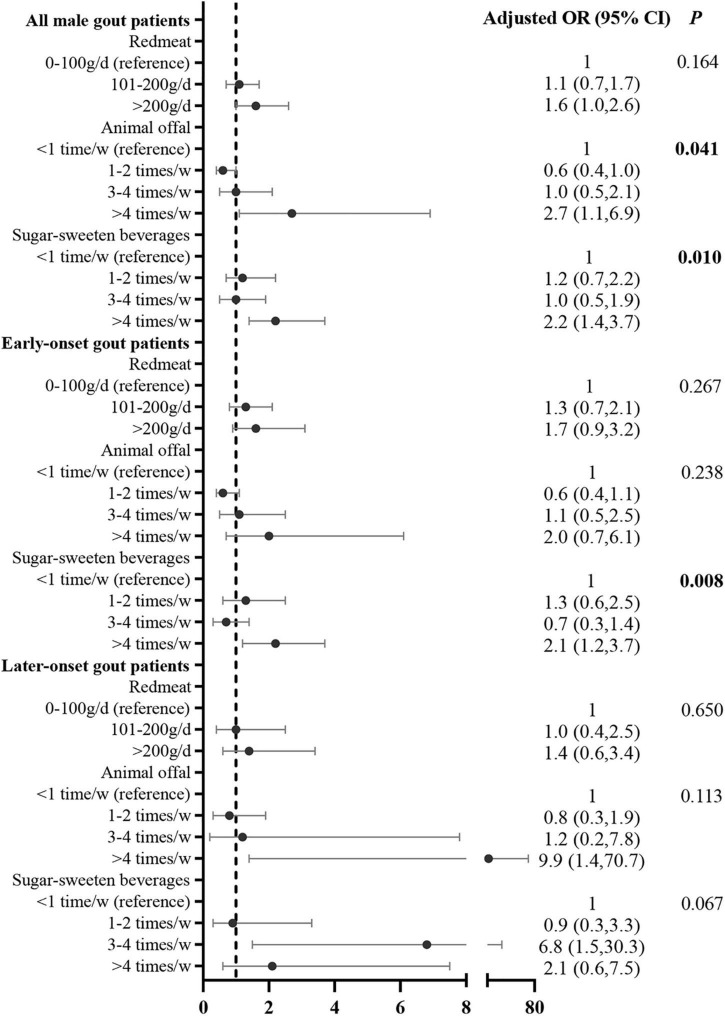
Correlation between food intake frequency with sUA ≥600 μmol/L in male gout patients after adjusting for potential confounders. Confounders included age, duration of gout, family history, BMI, eGFR, tophi, comorbidities (i.e., hypertension, T2DM, dyslipidemia, and coronary heart disease, current medication) (i.e., aspirin, diuretics, and lipid-lowering medication), and other dietary factors that were significant in the univariate analysis.

After adjusting for potential confounding variables, the multivariate logistic regression analysis showed that the risk factors for sUA level over 600 μmol/L were animal offal (compared with <once/week [reference], >4 times/week: adjusted OR = 2.7, 95% CI: 1.1, 6.9) and SSB consumption (compared with <once/week [reference], >4 times/week: adjusted OR = 2.2, 95% CI: 1.4, 3.7). The correlation between SSB consumption and sUA level over 600 μmol/L was significant in early onset gout patients (compared with <once/week [reference], >4 times/week: adjusted OR = 2.1, 95% CI: 1.2, 3.7; Figure 3). The correlation between SSB consumption and sUA level over 600 μmol/L was marginal (*p* = 0.062, Figure 3).

### Correlation Between Food Intake Frequency and Obesity

Similarly, the univariate logistic regression analysis for all male gout patients showed that red meat (compared with <100 g/day [reference], >200 g/day: OR = 2.0, 95% CI: 1.2, 3.3) and SSB consumption (compared with <once/week [reference], 1–2 times/week: OR = 2.5, 95% CI: 1.4, 4.4; 3–4 times/week: OR = 2.6, 95% CI: 1.4, 4.6; >4 times/week: OR = 2.9, 95% CI: 1.8, 4.7; Table 5) were positively correlated with obesity. In early onset gout patients, both red meat (compared with <100 g/day [reference], >200 g/day: OR = 2.0, 95% CI: 1.0, 3.5) and SSB consumption (compared with <once/week [reference], 1–2 times/week: OR = 2.5, 95% CI: 1.3, 4.8; 3–4 times/week: OR = 2.2, 95% CI: 1.1, 4.2; >4 times/week: OR = 2.4, 95% CI: 1.3, 4.2; Table 5) were positively correlated with obesity. For later-onset gout patients, the correlation between food intake frequency and obesity weren’t significant (*p* > 0.05).

**TABLE 5:** Univariate correlation between food intake frequency and obesity in male gout patients.

Food intake frequency	All male gout patients		Early onset gout patients		Later-onset gout patients	
	OR (95% CI)	*P*	OR (95% CI)	*P*	OR (95% CI)	*P*
**Alcohol**						
0–28 g/day	1	0.137	1	0.136	1	0.606
29–84 g/day	0.8 (0.4, 1.5)		1.0 (0.5, 2.0)		0.6 (0.1, 2.9)	
≥84 g/day	**0.5 (0.3, 1.0)**		**0.3 (0.1, 1.0)**		1.4 (0.5, 3.6)	
**Red meat**						
Less than 100 g/day	1	**0.008**	1	**0.016**	1	0.117
101–200 g/day	1.1 (0.7, 1.7)		0.8 (0.5, 1.5)		0.9 (0.3, 3.1)	
≥200 g/day	**2.0 (1.2, 3.3)**		**2.0 (1.0, 3.5)**		2.5 (0.9, 6.7)	
**Animal offal**						
Less than 1 time/week	1	0.251	1	0.284	1	0.493
1–2 times/week	1.0 (0.6, 1.6)		1.2 (0.7, 2.1)		0.3 (0.7, 1.3)	
3–4 times/week	1.3 (0.6, 2.6)		1.2 (0.5, 2.7)		0.7 (0.1, 6.1)	
>4 times/week	0.1 (0.0, 1.1)		0.2 (0.0, 1.2)		0.0 (0.0, 0.0)	
**Seafood**						
Less than 1 time/week	1	0.315	1	0.370	1	0.944
1–2 times/week	1.6 (1.0, 2.5)		1.6 (0.9, 2.8)		0.8 (0.2, 2.9)	
3–4 times/week	1.2 (0.6, 2.5)		1.2 (0.5, 3.0)		1.3 (0.4, 5.1)	
>4 times/week	0.9 (0.4, 2.1)		1.1 (0.5, 2.9)		0.0 (0.0, 0.0)	
**Hotpot**						
Less than 1 time/week	1	0.900	0	1.0	1	1.0
1–2 times/week	0.8 (0.4, 1.6)		1.0 (0.5, 2.2)		0.0 (0.0, 0.0)	
3–4 times/week	0.8 (0.2, 3.7)		1.0 (0.2, 5.4)		0.0 (0.0, 0.0)	
>4 times/week	0.0 (0.0, 0.0)		0.0 (0.0, 0.0)		0.0 (0.0, 0.0)	
**Slow-cooking soup**						
Less than 1 time/week	1	0.759	1	0.512	1	0.972
1–2 times/week	1.0 (0.7, 1.6)		0.9 (0.6, 1.6)		1.0 (0.4, 2.7)	
3–4 times/week	0.7 (0.4, 1.4)		0.6 (0.3, 1.2)		1.0 (0.2, 3.7)	
>4 times/week	0.9 (0.5, 1.8)		1.0 (0.4, 2.1)		0.7 (0.1, 3.3)	
**Sugar-sweeten beverages**						
Less than 1 time/week	1	***P* < 0.001**	1	**0.009**	1	0.870
1–2 times/week	**2.5 (1.4, 4.4)**		**2.5 (1.3, 4.8)**		0.9 (0.2, 4.2)	
3–4 times/week	**2.6 (1.4, 4.6)**		**2.2 (1.1, 4.2)**		1.0 (0.1, 8.0)	
>4 times/week	**2.9 (1.8, 4.7)**		**2.4 (1.3, 4.2)**		1.7 (0.5, 6.5)	
**Tea**						
Less than 1 time/week	1	0.430	1	0.189	1	0.709
1–2 times/week	0.7 (0.3, 1.4)		0.8 (0.4, 1.9)		0.3 (0.0, 2.6)	
3–4 times/week	1.6 (0.7, 3.3)		2.2 (0.9, 5.3)		0.5 (0.1, 4.7)	
>4 times/week	1.0 (0.7, 1.5)		1.4 (0.8, 2.3)		0.8 (0.3, 1.9)	
**Coffee**						
Less than 1 time/week	1	0.489	1	0.260	1	0.547
1–2 times/week	0.8 (0.3, 2.0)		0.8 (0.3, 2.0)		0.0 (0.0, 0.0)	
3–4 times/week	1.5 (0.3, 7.8)		0.7 (0.1, 6.0)		8.0 (0.5, 132.7)	
>4 times/week	2.0 (0.7, 5.7)		3.2 (0.9, 10.7)		0.0 (0.0, 0.0)	
**Milk and milk product**						
Less than 1 time/week	1	0.800	1	0.917	1	0.647
1–2 times/week	1.1 (0.7, 1.8)		1.0 (0.6, 1.7)		0.7 (0.2, 2.6)	
3–4 times/week	1.1 (0.5, 2.3)		0.8 (0.3, 2.0)		2.2 (0.5, 8.6)	
>4 times/week	0.7 (0.3, 1.6)		1.2 (0.5, 2.9)		0.0 (0.0, 0.0)	

*Bold values mean the difference is statistically significant.*

**FIGURE 4:**
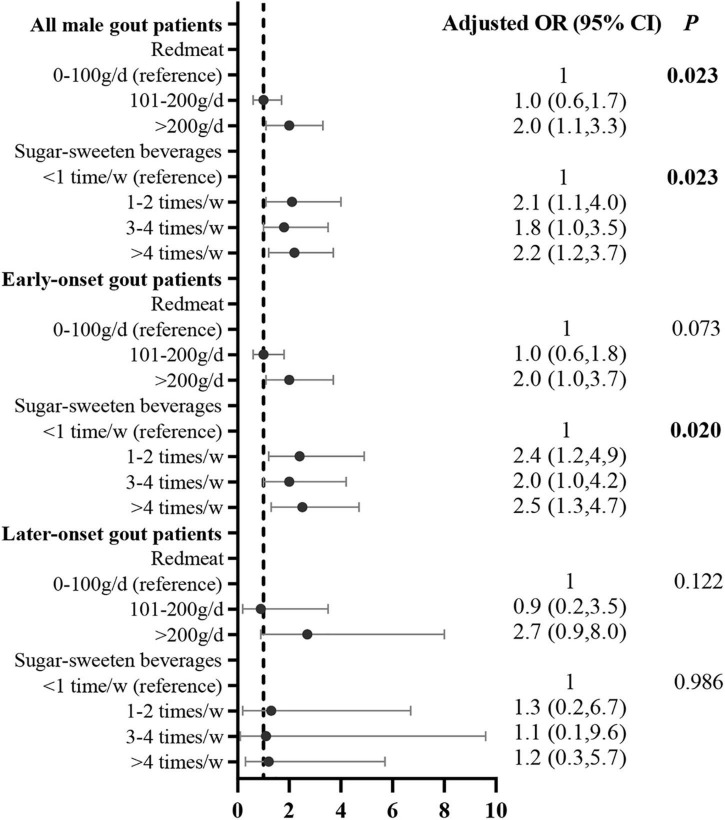
Correlation between food intake frequency with obesity in male gout patients after adjusting for potential confounders. Confounders included age, duration of gout, tophi, eGFR, comorbidities (i.e., hypertension, T2DM, dyslipidemia, and coronary heart disease, and other dietary factors that were significant in the univariate analysis.

After adjusting for potential confounding variables, the multivariate logistic regression analysis demonstrated that the risk factors for obesity were red meat (compared with <100 g/day [reference], >200 g/day: adjusted OR = 2.0, 95% CI: 1.1, 3.3) and SSB consumption (compared with <once/week [reference], 1–2 times/week: adjusted OR = 2.1, 95% CI: 1.1, 4.0; 3–4 times/week: adjusted OR = 1.8, 95% CI: 1.0, 3.5; >4 times/week: adjusted OR = 2.2, 95% CI: 1.2, 3.7). The correlation between SSB consumption and obesity was significant in early onset gout patients (compared with <once/week [reference], 1–2 times/week: adjusted OR = 2.4, 95% CI: 1.2, 4.9; 3–4 times/week: adjusted OR = 2.0, 95% CI: 1.0, 4.2; >4 times/week: adjusted OR = 2.5, 95% CI: 1.3, 4.7; Figure 4).

## Discussion

Our study showed that male gout patients with an early onset were characterized by a higher sUA level and a higher prevalence of obesity. For dietary habits before the onset of gout, early onset male gout patients consumed more SSB, red meat, and milk and milk product, but less alcohol and tea. In addition, a higher intake of SSB was associated with a higher sUA level and prevalence of obesity. Our results suggested that a higher sUA level and prevalence of obesity in male gout patients with an early onset is attributed to high SSB intake rather than other common gout-related dietary risk factors.

Both the prevalence of obesity and sUA level in the general population increase with age (26). Therefore, a higher prevalence of obesity and a higher sUA level in early onset than in later-onset male gout patients seems counter-intuitive. Obesity in youth has a detrimental effect on morbidity and mortality (26). Higher concentrations of sUA make it difficult to achieve a target sUA level. What’s more, sUA is a risk factor for hypertension, coronary heart disease, and atrial stiffness (1, 27, 28). Thus, higher blood uric acid levels and a greater prevalence of obesity negatively impact young gout patients. Therefore, these issues must be addressed and managed appropriately during the management of these gout patients.

Worldwide, there are significant differences in eating habits between generations. The China National Nutrition and Health Survey showed a trend of increased SSB consumption and decreased alcohol and tea consumption in youth (29, 30). In Norway, Australia, Netherlands, and the United States, young people consume more SSB but less wine and tea than older people (31–33). Dietary factors that contribute to the development of gout have shifted from excessive alcohol consumption in later-onset gout patients to excessive intake of SSB and red meat in early onset gout patients.

SSB can increase the sUA level by depleting adenosine triphosphate during fructose metabolism, which, in turn, leads to the accumulation of adenosine monophosphate (AMP). The lack of free phosphate results in the conversion of AMP to the uric acid precursor inosine monophosphate (34). In prospective cohort studies, consumption of one serving per day of sugar-sweetened soft drinks led to a 45 and 74% higher gout risk in men (6) and women, respectively, than those who consumed less than one serving per month (5). In European ancestor gout patients, early onset gout patients consumed more SSB than later-onset gout patients (16). Similarly, our data revealed that early onset gout patients consumed more SSB and had a significantly higher sUA level than later-onset gout patients. Moreover, SSB consumption in male patients was positively correlated with sUA level over 600 μmol/L. Thus, higher SSB consumption may contribute to a paradoxically higher sUA level in younger gout patients with a higher eGFR.

A meta-analysis showed that intake of SSB increased the risk of being overweight or obese by 55% among groups with the highest intake compared with those with the lowest intake (35). Obesity increased sUA level and risk of developing gout. The United States National Health and Nutrition Examination Surveys conducted from 1988 to 1994 and 2007 to 2010 revealed that each unit increase in BMI was associated with a 5% higher prevalence of gout, even after adjustment for sUA level (36). Furthermore, patients with obesity are 2.24 times more likely to develop gout (37), and a prospective cohort study showed that obesity is associated with earlier onset of gout (38). Our study showed that SSB intake was positively correlated with obesity in male gout patients and early onset gout patients. Therefore, greater SSB intake likely contributes to a higher rate of obesity in early onset gout patients.

The correlation between intergenerational changes in dietary habits and intergenerational changes in clinical features of gout has been overlooked in previous studies (11–16). To our knowledge, the present study firstly demonstrated that of the many dietary factors, only SSB is associated with elevated sUA level and higher obesity rate in patients with early onset gout. In patients with early onset gout, attention should be paid to finding unfavorable dietary habits, with particular attention to SSB intake and advice to reduce added sugar intake. Dietary and exercise advice should be given to obese patients with early onset gout. However, reducing SSB intake cannot be left only to clinicians. The sugar industry has driven the growth of the SSB trend *via* pervasive marketing, which has influenced government policy, scientific research, and the diets of the general population (39). Thus, reducing the intake of SSB requires public health measures. The impact of introducing a sugar tax on the incidence of gout in the United States has been modeled, which showed that it could prevent almost 85,000 gout cases over 15 years, save more than 25,000 quality-adjusted life-years, and $3 billion (40).

There are several limitations to this study. First, this was a single-center study, which may have selection bias. Thus, our data from a university-affiliated hospital requires further confirmation using data from other centers. However, the patient characteristics of our study are similar to those of a study conducted at another university-affiliated hospital in China (13). Second, we investigated food intake frequency before the first gout attack. In the present study, patients had difficulty recalling the ingredients of the SSB and therefore could not calculate amount of fructose intake in detail. What’s more, recall bias may be present in some patients, especially those with long disease duration. However, previous studies have suggested that past diets of up to 10 years prior are recalled with acceptable accuracy (41, 42). In this study, the median duration of gout was 3 years, and most patients had had gout for less than 10 years. Third, our study didn’t include healthy population. Therefore, we couldn’t explore the difference of gout related dietary habits between gout patients and healthy population. Fourth, total energy intake and physical activity of participants which were unavailable in the present study might be act as potential confounders. Differences in total energy intake and physical activity between early onset group and later-onset group is an interesting issue in future research. Lastly, our study assessed the relationship between dietary habits during the 1 year prior to an initial gout attack, current blood uric acid level, and obesity. A multicenter prospective study of a dynamic dietary survey on a representative community population should be conducted in the future which can assess whether dietary habits contribute to gout earlier onset, correlate with different gout clusters (43) and increase healthcare and economic burden of gout.

In conclusion, our study showed that the dietary factors that contribute to the development of gout have shifted from excessive alcohol consumption in later-onset gout patients to excessive SSB intake in early onset gout patients. This change was associated with a higher prevalence of obesity and higher sUA level in early onset gout patients. Thus, clinicians should provide specific dietary education for early onset and later-onset gout patients. The epidemic of excessive SSB consumption should be considered for the development of public health policies for the prevention of gout.

## Data Availability Statement

The datasets presented in this article are not readily available because disclosure of raw data requires regulatory approval. Requests to access the datasets should be directed to LD, dailie@mail.sysu.edu.cn.

## Ethics Statement

The studies involving human participants were reviewed and approved by the Ethics Committee of Sun Yat-sen Memorial Hospital. Written informed consent to participate in this study was provided by the participants’ legal guardian/next of kin.

## Author Contributions

Q-HL, Y-WZ, and LD: conception, design, and data analysis and interpretation. LD: administrative support. Q-HL, J-JL, Y-QM, and LD: provision of study materials or patients. S-YL, Y-FB, CD, and K-MY: collection and assembly of data. Q-HL and Y-WZ: writing the draft of the manuscript. All authors take part in draft revising and final approval of the manuscript.

## Conflict of Interest

The authors declare that the research was conducted in the absence of any commercial or financial relationships that could be construed as a potential conflict of interest.

## Publisher’s Note

All claims expressed in this article are solely those of the authors and do not necessarily represent those of their affiliated organizations, or those of the publisher, the editors and the reviewers. Any product that may be evaluated in this article, or claim that may be made by its manufacturer, is not guaranteed or endorsed by the publisher.
